# Meet the Section Editor

**DOI:** 10.2174/1570159X2108230510125436

**Published:** 2023-06-15

**Authors:** Sergey Bachurin

**Affiliations:** 1 Institute of Physiologically Active Compounds Russian Academy of Sciences (IPAC RAS), Chernogolovka, Russian Federation

Professor Sergey Bachurin received the Ph.D. degree in 1980 from Moscow State University. From 2006 to 2018, he remained the Director of the Institute of Physiologically active Compounds RAS. In 2003, he was elected as a member of the Russian Academy of Sciences. His research activity is related to the discovery of novel agents for neurodegenerative diseases treatment, in particular for Alzheimer's disease. He is the author of more than 250 articles in peer-reviewing journals and about 40 patents. His h-index is 33. Prof. Bachurin was an invited lecturer at the University of California (San Francisco), and at Tufts University (Boston).
